# Neonatal complications and risk factors associated with assisted vaginal delivery

**DOI:** 10.1038/s41598-024-62703-x

**Published:** 2024-05-25

**Authors:** Saifon Chawanpaiboon, Vitaya Titapant, Julaporn Pooliam

**Affiliations:** 1 Division of Maternal-Fetal Medicine, Department of Obstetrics and Gynecology, Faculty of Medicine Siriraj Hospital, Mahidol University, Bangkok, 10700 Thailand; 2 Clinical Epidemiological Unit, Office for Research and Development, Faculty of Medicine Siriraj Hospital, Mahidol University, Bangkok, 10700 Thailand

**Keywords:** Assisted, Forceps, Neonatal complication, Vacuum, Vaginal delivery, Paediatric research, Neonatology

## Abstract

To investigate neonatal injuries, morbidities and risk factors related to vaginal deliveries. This retrospective, descriptive study identified 3500 patients who underwent vaginal delivery between 2020 and 2022. Demographic data, neonatal injuries, complications arising from vaginal delivery and pertinent risk factors were documented. Neonatal injuries and morbidities were prevalent in cases of assisted vacuum delivery, gestational diabetes mellitus class A2 (GDMA2) and pre-eclampsia with severe features. Caput succedaneum and petechiae were observed in 291/3500 cases (8.31%) and 108/3500 cases (3.09%), respectively. Caput succedaneum was associated with multiparity (adjusted odds ratio [AOR] 0.36, 95% confidence interval [CI] 0.22–0.57, *P* < 0.001) and assisted vacuum delivery (AOR 5.18, 95% CI 2.60–10.3, *P* < 0.001). Cephalohaematoma was linked to GDMA2 (AOR 11.3, 95% CI 2.96–43.2, *P* < 0.001) and assisted vacuum delivery (AOR 16.5, 95% CI 6.71–40.5, *P* < 0.001). Scalp lacerations correlated with assisted vacuum and forceps deliveries (AOR 6.94, 95% CI 1.85–26.1, *P* < 0.004; and AOR 10.5, 95% CI 1.08–102.2, *P* < 0.042, respectively). Neonatal morbidities were associated with preterm delivery (AOR 3.49, 95% CI 1.39–8.72, *P* = 0.008), night-time delivery (AOR 1.32, 95% CI 1.07–1.63, *P* = 0.009) and low birth weight (AOR 7.52, 95% CI 3.79–14.9, *P* < 0.001). Neonatal injuries and morbidities were common in assisted vacuum delivery, maternal GDMA2, pre-eclampsia with severe features, preterm delivery and low birth weight. Cephalohaematoma and scalp lacerations were prevalent in assisted vaginal deliveries. Most morbidities occurred at night.

Clinical trial registration: Thai Clinical Trials Registry 20220126004.

## Introduction

Adverse events related to vaginal delivery are uncommon, given that delivery is a natural process that can occur spontaneously. However, there are instances where assisted vaginal delivery becomes necessary due to labour difficulties. Factors such as the duration of labour and its phase (latent or active) can influence delivery outcomes. Before attending to delivery, it is crucial to consider potential birth injuries, complications and relevant factors. A birth injury can be linked to an impairment in the neonate’s bodily function or structure. Such injuries often occur during labour, delivery or post-delivery, particularly in neonates needing resuscitation.

Vaginal delivery can either be spontaneous or assisted. Spontaneous vaginal delivery occurs when a pregnant woman goes into labour without requiring drugs or techniques to induce labour and delivery^1^. Assisted vaginal delivery involves using instruments such as forceps or a vacuum to aid delivery^2^.

Assisted vaginal births are performed to expedite birth for the benefit of both mothers and babies but can occasionally be associated with morbidity for both parties. Indications for assisted vaginal delivery include a prolonged second stage of labour, suspicion of fetal compromise, or the need to shorten the second stage of labour for maternal benefit^3^. The decision to use instruments such as forceps or vacuum extraction often hinges on their availability, the clinical circumstances, and the obstetrician’s preference and experience. Assisted vaginal delivery is crucial for mothers facing challenging labour. Both forceps assistance and vacuum extraction should only be administered by obstetricians with ample experience to minimise potential complications. In the United States, 3.1% of all births in 2017 occurred via an assisted vaginal approach^4^. Forceps-assisted births constituted 0.5% of vaginal deliveries, while vacuum-assisted ones accounted for 2.6%. The prevalence of assisted vaginal births varied both within and across US regions^4^, suggesting that the choice of method may depend on practitioners’ familiarity and expertise^5^. Overall, rates of assisted vaginal births have declined both nationally and regionally in the United States^5^.

However, complications and unexpected events can still arise from vaginal deliveries. Our study investigated complications in newborns resulting from vaginal deliveries by reviewing the details of complications encountered at Siriraj Hospital. We also explored the risk factors related to complications.

## Methods

This retrospective study was conducted within the statistical unit of the Department of Obstetrics and Gynaecology at the Faculty of Medicine Siriraj Hospital. Prior to commencing the research, we secured approval from the Ethics Committee of the Faculty of Medicine Siriraj Hospital (approval number Si 185/2022) and registered the study in the Thai Clinical Trials Registry (TCTR 20220126004).

Data regarding pregnant women who underwent caesarean sections between 2020 and 2022 were sourced from hospital records. We identified a total of 3500 cases. Neonatal injuries and complications stemming from vaginal deliveries at Siriraj Hospital were documented.

We employed multivariable analysis to determine the factors contributing to neonatal cephalic injuries and neonatal complications. These factors were identified based on their significance in univariate analysis. For neonatal cephalic injuries, the variables considered included the type of vaginal delivery, maternal health conditions such as hypertension (HT) and diabetes mellitus (DM), the prolongation of the second stage of labor, the surgeon who performed delivery, the time of delivery and gestational age at delivery. For neonatal complications, significant variables from univariate analysis comprised the type of vaginal delivery, maternal health conditions (HT, DM), prolongation of the second stage of labor, the surgeon who performed delivery, gestational age at delivery, birth weight, and time of delivery.

### Statistical analysis

Demographic data were compiled using descriptive statistics. Categorical data are presented as numbers and percentages, while continuous data are reported as the means ± standard deviations or medians and ranges. We employed PASW Statistics (version 18; SPSS Inc, Chicago, IL, USA) for our statistical analyses. Baseline data (qualitative parameters, maternal complications and infant complications arising from caesarean section) were compared using the chi-squared and Fisher’s exact tests. For quantitative variables, the Mann–Whitney *U* test was employed for univariate analysis, while multiple logistic regression was utilised for multivariate analysis.

### Terminology

The following terms were used in this study^6^:

- Bruising: this occurs when capillaries rupture, allowing blood cells to seep deep beneath the skin, resulting in the characteristic ‘black and blue’ marks on the skin. Over time, as the body metabolises the substances in the blood cells, the bruise changes colour, potentially appearing purple, brown, or even green.
- Petechiae: this involves bleeding under the skin, characterised by marks resembling a rash of small dots. Petechiae occurs when tiny blood vessels break open, causing blood to leak into the skin and giving it the appearance of a rash.
- Caput succedaneum: this refers to scalp swelling that occurs during labour and is usually evident shortly after delivery. It is commonly associated with prolonged pressure on the fetal head during delivery or a prolonged second stage of labour.
- Cephalohaematoma: this condition involves a subperiosteal collection of blood that occurs due to the rupture of vessels beneath the periosteum, typically over the parietal or occipital bone. It presents as swelling that does not cross suture lines.
- Subgaleal haematoma: this refers to the accumulation of blood in the loose areolar tissue between the periosteum of the skull and the aponeurosis.

### Ethics approval and consent to participate

Prior to the commencement of this study, the requisite ethical clearance was procured from the Siriraj Ethics Committee of the Faculty of Medicine, Siriraj Hospital (Si 185/2022). Additionally, this research was registered with the Thai Clinical Trials Registry (20220126004). This study was a retrospective chart review and informed consent was not required.

## Results

We retrieved data for 3500 neonates born through vaginal deliveries. For assisted vaginal deliveries, vacuum assistance was associated with significant increases in neonatal jaundice (63/221 [29.86%], *P* < 0.001), caput succedaneum (87/221 [41.23%], *P* < 0.001), cephalohaematoma (35/221 [16.59%], *P* < 0.001) and subgaleal haematoma (11/221 [8.06%], *P* < 0.001; Table 1).

**Table 1:** Relationship between neonatal injuries, morbidities and types of vaginal delivery (univariate analysis).

Complications	All (N = 3500)	Vaginal delivery n (%)			*P*
		Spontaneous (n = 3271)	Vacuum extraction (n = 221)	Forceps assistant/extraction (n = 12)	
Caput succedaneum	291 (8.31%)	202 (6.18%)	87 (41.23%)	2 (16.67%)	< 0.001
Petechia	108 (3.09%)	107 (3.27%)	0 (0.00%)	0 (0.00%)	0.011
Jaundice	507 (14.49%)	443 (13.54%)	63 (29.86%)	0 (0.00%)	< 0.001
Bruise	90 (2.57%)	85 (2.60%)	4 (1.90%)	1 (8.33%)	0.543
Abrasion	64 (1.83%)	57 (1.74%)	2 (0.95%)	5 (41.67%)	< 0.001
Cephalohaematoma	79 (2.26%)	44 (1.35%)	35 (16.59%)	0 (0.00%)	< 0.001
Scalp laceration	49 (1.40%)	38 (1.16%)	10 (4.74%)	1 (8.33%)	< 0.001
Subgaleal haematoma	24 (0.69%)	7 (0.21%)	17 (8.06%)	0 (0.00%)	< 0.001
Brachial plexus injuries	4 (0.11%)	3 (0.09%)	1 (0.47%)	0 (0.00%)	0.465
Fractured clavicle	25 (0.71%)	21 (0.64%)	4 (1.90%)	0 (0.00%)	0.210
Ventilatory support	21 (0.60%)	20 (0.61%)	1 (0.47%)	0 (0.00%)	0.982
Postnatal adaptation	101 (2.89%)	89 (2.72%)	10 (4.74%)	1 (8.33%)	0.041
Birth asphyxia					
Mild	154 (4.40%)	115 (3.52%)	36 (17.06%)	0 (0.00%)	< 0.001
Severe	11 (0.31%)	8 (0.24%)	2 (0.95%)	0 (0.00%)	< 0.001
Respiratory distress syndrome	14 (0.40%)	11 (0.34%)	3 (1.42%)	0 (0.00%)	0.115
Transient tachypnea of the newborn	153 (4.37%)	137 (4.19%)	15 (7.11%)	0 (0.00%)	0.080
Pneumothorax	8 (0.23%)	7 (0.21%)	1 (0.47%)	0 (0.00%)	0.890
Desaturation	10 (0.29%)	9 (0.28%)	1 (0.47%)	0 (0.00%)	0.955
Pneumonia	9 (0.26%)	9 (0.28%)	0 (0.00%)	0 (0.00%)	0.889
Hypocalcaemia	6 (0.17%)	5 (0.15%)	1 (0.47%)	0 (0.00%)	0.747
Hypoglycaemia	51 (1.46%)	46 (1.41%)	5 (2.37%)	0 (0.00%)	0.671
Hypothermia	31 (0.89%)	29 (0.89%)	1 (0.47%)	1 (8.33%)	0.045
Sepsis	26 (0.74%)	24 (0.73%)	2 (0.95%)	0 (0.00%)	0.968
Death	5 (0.14%)	5 (0.15%)	0 (0.00%)	0 (0.00%)	0.950
Other	43 (1.23%)	39 (1.19%)	4 (1.90%)	0 (0.00%)	0.793
Total	1348 (38.51%)	1166 (35.6%)	170 (80.6%)	8 (66.7%)	< 0.001

The most common neonatal complications observed were neonatal jaundice (507/3500, 14.5%), caput succedaneum (291/3500, 8.3%) and transient tachypnea of the newborn (153/3500, 4.4%; Table 2).

**Table 2:** Overview of neonatal injuries and morbidities following vaginal delivery (multiple complications possible).

Details	Number of neonates (n)	Percentage (%)
Caput succedaneum	291	8.3
Neonatal jaundice	507	14.5
Neonatal injuries		
Bruise	90	2.6
Cephalohaematoma	79	2.3
Abrasion	64	1.8
Scalp laceration	49	1.4
Subgaleal hematoma	24	0.7
Fractured clavicle	25	0.7
Brachial plexus injury	4	0.1
Neonatal morbidities		
Birth asphyxia		
Mild	154	4.4
Severe	11	0.3
Transient tachypnea of the newborn	153	4.4
Ventilatory support	21	0.6
Respiratory distress syndrome	14	0.4
Pneumothorax	8	0.2
Desaturation	10	0.3
Pneumonia	9	0.3
Hypoglycaemia	51	1.5
Hypothermia	31	0.9
Hypocalcaemia	6	0.2
Neonatal sepsis	26	0.7
Neonatal death	5	0.1
Other	43	1.2
Total	1348	38.5

When comparing complications between primigravida and multiparity, it was found that neonatal jaundice (16.9%/12.3%, *P* < 0.001), mild birth asphyxia (5.17%/3.70%, *P* = 0.034), caput succedaneum (12.75%/4.3%, *P* < 0.001), cephalohaematoma (2.89%/1.69%, *P* = 0.017), subgaleal haematoma (1.14%/0.27%, *P* = 0.002) and total neonatal complications (43.42%/34.08%, *P* < 0.001) were higher in the primigravida group (Table 3).

**Table 3:** Relationship between neonatal injuries, morbidities and maternal parity (univariate analysis).

	All (N = 3500)	Parity		*P*
		0 (n = 1663)	≥ 1 (n = 1837)	
Caput succedaneum	291 (8.31%)	212 (12.75%)	79 (4.30%)	< 0.001
Petechia	108 (3.09%)	38 (2.29%)	70 (3.81%)	0.009
Jaundice	507 (14.49%)	281 (16.90%)	226 (12.30%)	< 0.001
Neonatal injuries				
Fractured clavicle	25 (0.71%)	7 (0.42%)	18 (0.98%)	0.050
Bruise	90 (2.57%)	40 (2.41%)	50 (2.72%)	0.555
Abrasion	64 (1.83%)	36 (2.16%)	28 (1.52%)	0.158
Cephalohaematoma	79 (2.26%)	48 (2.89%)	31 (1.69%)	0.017
Scalp laceration	49 (1.40%)	26 (1.56%)	23 (1.25%)	0.434
Subgaleal haematoma	24 (0.69%)	19 (1.14%)	5 (0.27%)	0.002
Brachial plexus	4 (0.11%)	2 (0.12%)	2 (0.11%)	1.000
Complications				
Birth asphyxia				
Mild	154 (4.40%)	86 (5.17%)	68 (3.70%)	0.034
Severe	11 (0.31%)	5 (0.30%)	6 (0.33%)	1.000
Ventilatory support	21 (0.60%)	11 (0.66%)	10 (0.54%)	0.654
Postnatal adaptation	101 (2.89%)	50 (3.01%)	51 (2.78%)	0.684
Transient tachypnea of the newborn	153 (4.37%)	71 (4.27%)	82 (4.46%)	0.779
Pneumothorax	8 (0.23%)	2 (0.12%)	6 (0.33%)	0.293
Desaturation	10 (0.29%)	6 (0.36%)	4 (0.22%)	0.533
Pneumonia	9 (0.26%)	4 (0.24%)	5 (0.27%)	1.000
Respiratory distress syndrome	14 (0.40%)	8 (0.48%)	6 (0.33%)	0.594
Hypothermia	31 (0.89%)	12 (0.72%)	19 (1.03%)	0.324
Hypoglycaemia	51 (1.46%)	26 (1.56%)	25 (1.36%)	0.618
Hypocalcaemia	6 (0.17%)	4 (0.24%)	2 (0.11%)	0.432
Sepsis	26 (0.74%)	16 (0.96%)	10 (0.54%)	0.151
Death	5 (0.14%)	3 (0.18%)	2 (0.11%)	0.673
Other	43 (1.23%)	16 (0.96%)	27 (1.47%)	0.173
Total	1348 (38.51%)	722 (43.42%)	626 (34.08%)	< 0.001

Among mothers with gestational diabetes mellitus, those with class A2 (GDMA2) had a higher incidence of mild birth asphyxia (3/21 [14.29%], *P* = 0.028), cephalohaematoma (3/21 [14.29%], *P* = 0.028), scalp laceration (2/21 [(9.52%], *P* = 0.007) and total neonatal complications (13/21 [61.9%], *P* < 0.01; Table 4).

**Table 4:** Correlation of neonatal injuries and morbidities with maternal diabetes mellitus and hypertension (univariate analysis).

Complications	All (N = 3500)	Maternal gestational diabetes (GDM) during pregnancy n (%)				*P*	Hypertension (HT) n (%)				*P*	
		No (n = 3084)	GDMA1 (n = 377)	GDMA2 (n = 21)	Pre-existing diabetes mellitus (n = 18)		No (n = 3259)	Gestational HT/transient (n = 198)	Mild to severe pre-eclampsia (n = 16)	Pre-existing HT (n = 27)		
Caput succedaneum	291 (8.31%)	255 (8.27%)	33 (8.75%)	1 (4.76%)	2 (11.11%)	0.888	263 (8.07%)	24 (12.12%)	2 (12.50%)	2 (7.41%)	0.220	
Petechia	108 (3.09%)	84 (2.72%)	23 (6.10%)	0 (0.0%)	1 (5.56%)	0.003	98 (3.01%)	9 (4.55%)	0 (0.0%)	1 (3.70%)	0.568	
Jaundice	507 (14.49%)	449 (14.56%)	53 (14.06%)	3 (14.29%)	2 (11.11%)	0.972	460 (14.11%)	39 (19.70%)	3 (18.75%)	5 (18.52%)	0.152	
Neonatal injuries												
Fractured clavicle	25 (0.71%)	22 (0.71%)	3 (0.80%)	0 (0.0%)	0 (0.0%)	0.957	21 (0.64%)	4 (2.02%)	0 (0.0%)	0 (0.0%)		0.151
Bruise	90 (2.57%)	76 (2.46%)	14 (3.71%)	0 (0.0%)	0 (0.0%)	0.372	85 (2.61%)	4 (2.02%)	0 (0.0%)	1 (3.70%)		0.845
Abrasion	64 (1.83%)	54 (1.75%)	8 (2.12%)	1 (4.76%)	1 (5.56%)	0.443	61 (1.87%)	2 (1.01%)	0 (0.0%)	1 (3.70%)		0.659
Cephalohaematoma	79 (2.26%)	66 (2.14%)	10 (2.56%)	3 (14.29%)	0 (0.00%)	0.002	72 (2.21%)	5 (2.53%)	0 (0.0%)	2 (7.41%)		0.294
Scalp laceration	49 (1.40%)	39 (1.26%)	8 (2.12%)	2 (9.52%)	0 (0.0%)	0.007	42 (1.29%)	5 (2.53%)	0 (0.0%)	2 (7.41%)		0.024
Subgaleal haematoma	24 (0.69%)	19 (0.62%)	5 (1.33%)	0 (0.0%)	0 (0.0%)	0.430	21 (0.64%)	2 (1.01%)	0 (0.0%)	1 (3.70%)		0.250
Brachial plexus injury	4 (0.11%)	4 (0.13%)	0 (0.0%)	0 (0.0%)	0 (0.0%)	0.910	4 (0.12%)	0 (0.0%)	0 (0.0%)	0 (0.0%)		0.961
Neonatal morbidities												
Pneumothorax	8 (0.23%)	6 (0.19%)	1 (0.27%)	1 (4.76%)	0 (0.0%)	< 0.001	6 (0.18%)	1 (0.51%)	0 (0.0%)	1 (3.70%)		0.002
Desaturation	10 (0.29%)	9 (0.29%)	0 (0.0%)	1 (4.76%)	0 (0.0%)	0.001	9 (0.28%)	1 (0.51%)	0 (0.0%)	0 (0.0%)		0.926
Pneumonia	9 (0.26%)	9 (0.29%)	0 (0.0%)	0 (0.0%)	0 (0.0%)	0.749	9 (0.28%)	0 (0.0%)	0 (0.0%)	0 (0.0%)		0.881
Ventilatory support	21 (0.60%)	17 (0.55%)	4 (1.06%)	0 (0.0%)	0 (0.0%)	0.637	21 (0.64%)	0 (0.0%)	0 (0.0%)	0 (0.0%)		0.668
Postnatal adaptation	101 (2.89%)	96 (3.11%)	4 (1.06%)	1 (4.76%)	0 (0.0%)	0.119	95 (2.92%)	6 (3.03%)	0 (0.0%)	0 (0.0%)		0.729
Birth asphyxia												
Mild	154 (4.40%)	126 (4.09%)	24 (6.37%)	3 (14.29%)	1 (5.56%)	0.028	145 (4.45%)	6 (3.03%)	1 (6.25%)	2 (7.41%)		0.657
Severe	11 (0.31%)	11 (0.36%)	0 (0.0%)	0 (0.0%)	0 (0.0%)	0.685	10 (0.31%)	1 (0.51%)	0 (0.0%)	0 (0.0%)		0.941
Transient tachypnea of the newborn	153 (4.37%)	131 (4.25%)	19 (5.04%)	2 (9.52%)	1 (5.56%)	0.591	136 (4.17%)	14 (7.07%)	3 (18.75%)	0 (0.0%)		0.005
Respiratory distress syndrome	14 (0.40%)	13 (0.42%)	1 (0.27%)	0 (0.0%)	0 (0.0%)	0.948	13 (0.40%)	0 (0.0%)	1 (6.25%)	0 (0.0%)		0.002
Hypothermia	31 (0.89%)	30 (0.97%)	1 (0.27%)	0 (0.0%)	0 (0.0%)	0.519	30 (0.92%)	1 (0.51%)	0 (0.0%)	0 (0.0%)		0.860
Hypoglycaemia	51 (1.46%)	43 (1.39%)	6 (1.59%)	1 (4.76%)	1 (5.56%)	0.280	45 (1.38%)	3 (1.52%)	2 (12.50%)	1 (3.70%)		0.002
Hypocalcaemia	6 (0.17%)	6 (0.19%)	0 (0.0%)	0 (0.0%)	0 (0.0%)	0.847	5 (0.15%)	0 (0.0%)	1 (6.25%)	0 (0.0%)		< 0.001
Sepsis	26 (0.74%)	21 (0.68%)	4 (1.06%)	0 (0.0%)	1 (5.56%)	0.090	24 (0.74%)	1 (0.51%)	1 (6.25%)	0 (0.0%)		0.074
Death	5 (0.14%)	4 (0.13%)	1 (0.27%)	0 (0.0%)	0 (0.0%)	0.921	4 (0.12%)	1 (0.51%)	0 (0.0%)	0 (0.0%)		0.578
Other	43 (1.23%)	38 (1.23%)	5 (1.33%)	0 (0.0%)	0 (0.0%)	0.916	39 (1.20%)	3 (1.52%)	1 (6.25%)	0 (0.0%)		0.281
Total	1348 (38.51%)	1162 (37.68%)	167 (44.30%)	13 (61.90%)	6 (33.33%)	0.010	1239 (38.02%)	89 (44.95%)	10 (62.50%)	10 (37.04%)		0.052

In mothers with hypertension, those with mild to severe pre-eclampsia showed elevated rates of hypoglycaemia (2/16 [12.50%], *P* = 0.002), transient tachypnoea (3/16 [18.75%], respiratory distress syndrome (1/16 [6.25%], *P* = 0.002) and hypocalcaemia (1/16 [6.25%], *P* < 0.001). However, the total number of neonatal complications was not significantly different (Table 4).

Neonates born preterm had significantly more complications than those born at term. The complications were a need for ventilator support (5.77%/0.44%, *P* < 0.001), neonatal jaundice (38.46%/13.75%, *P* < 0.001), hypoglycaemia (7.69%/1.27%, *P* < 0.001), sepsis (2.88%/0.68%, *P* = 0.040), respiratory distress syndrome (2.88%/0.32%, *P* = 0.007), hypocalcaemia (1.92%/0.12%, *P* = 0.012), and skin abrasion (7.69%/1.65%, *P* < 0.001). The total complication rates were 59.62% for neonates born preterm and 37.87% for those born at term (*P* < 0.001; Table 5).

**Table 5:** Correlation of neonatal injuries and morbidities with gestational age at the time of delivery (univariate analysis).

Complications	All (N = 3500)	Gestational age at delivery (weeks of gestation) n (%)		*P*
		Preterm delivery (< 37) (n = 104)	Term delivery (≥ 37) (n = 3396)	
Caput succedaneum	291 (8.31%)	8 (7.69%)	283 (8.33%)	0.816
Petechia	108 (3.09%)	2 (1.92%)	106 (3.12%)	0.771
Neonatal injury				
Bruise	90 (2.57%)	1 (0.96%)	89 (2.62%)	0.523
Skin abrasion	64 (1.83%)	8 (7.69%)	56 (1.65%)	< 0.001
Cephalohaematoma	79 (2.26%)	3 (2.88%)	76 (2.24%)	0.509
Scalp laceration	49 (1.40%)	1 (0.96%)	48 (1.41%)	1.000
Subgaleal haematoma	24 (0.69%)	1 (0.96%)	23 (0.68%)	0.516
Brachial plexus injury	4 (0.11%)	0 (0.00%)	4 (0.12%)	1.000
Fractured clavicle	25 (0.71%)	0 (0.00%)	25 (0.74%)	1.000
Neonatal morbidities				
Ventilatory support	21 (0.60%)	6 (5.77%)	15 (0.44%)	< 0.001
Postnatal adaptation	101 (2.89%)	1 (0.96%)	100 (2.94%)	0.370
Birth asphyxia				
Mild	154 (4.40%)	5 (4.81%)	149 (4.39%)	0.806
Severe	11 (0.31%)	1 (0.96%)	10 (0.29%)	0.283
Transient tachypnea of the newborn	153 (4.37%)	8 (7.69%)	145 (4.27%)	0.135
Respiratory distress syndrome	14 (0.40%)	3 (2.88%)	11 (0.32%)	0.007
Pneumothorax	8 (0.23%)	0 (0.00%)	8 (0.24%)	1.000
Desaturation	10 (0.29%)	1 (0.96%)	9 (0.27%)	0.261
Pneumonia	9 (0.26%)	1 (0.96%)	8 (0.24%)	0.238
Hypothermia	31 (0.89%)	3 (2.88%)	28 (0.82%)	0.063
Hypocalcaemia	6 (0.17%)	2 (1.92%)	4 (0.12%)	0.012
Hypoglycaemia	51 (1.46%)	8 (7.69%)	43 (1.27%)	< 0.001
Sepsis	26 (0.74%)	3 (2.88%)	23 (0.68%)	0.040
Death	5 (0.14%)	0 (0.00%)	5 (0.15%)	1.000
Other	43 (1.23%)	6 (5.77%)	37 (1.09%)	0.001
Neonatal complications	1348 (38.51%)	62 (59.62%)	1286 (37.87%)	< 0.001

Neonates born to mothers with a prolonged second stage of labour had significantly higher rates of complications (*P* < 0.001). The complications were neonatal jaundice (26.73%), mild birth asphyxia (13.86%), caput succedaneum (38.61%), cephalohaematoma (17.82%), scalp laceration (5.94%) and subgaleal haematoma (9.9%; Table 6).

**Table 6:** Association of neonatal injuries and morbidities with prolonged second stage of labour and fetal distress (univariate analysis).

	All (N = 3500)	Prolonged second stage of labour		*P*	Fetal distress		*P*
		No (n = 3399)	Yes (n = 101)		No (n = 3355)	Yes (n = 145)	
Caput succedaneum	291 (8.31%)	252 (7.41%)	39 (38.61%)	< 0.001	225 (6.71%)	66 (45.52%)	< 0.001
Petechia	108 (3.09%)	108 (3.18%)	0 (0.00%)	0.075	108 (3.22%)	0 (0.00%)	0.028
Jaundice	507 (14.49%)	480 (14.12%)	27 (26.73%)	< 0.001	463 (13.80%)	44 (30.34%)	< 0.001
Neonatal injuries							
Cephalohaematoma	79 (2.26%)	61 (1.79%)	18 (17.82%)	< 0.001	58 (1.73%)	21 (14.48%)	< 0.001
Scalp laceration	49 (1.40%)	43 (1.27%)	6 (5.94%)	0.003	45 (1.34%)	4 (2.76%)	0.143
Subgaleal haematoma	24 (0.69%)	14 (0.41%)	10 (9.90%)	< 0.001	13 (0.39%)	11 (7.59%)	< 0.001
Brachial plexus injury	4 (0.11%)	4 (0.12%)	0 (0.00%)	1.000	3 (0.09%)	1 (0.69%)	0.156
Birth injury/fracture	25 (0.71%)	24 (0.71%)	1 (0.99%)	0.520	22 (0.66%)	3 (2.07%)	0.082
Neonatal morbidities							
Ventilatory support	21 (0.60%)	21 (0.62%)	0 (0.00%)	1.000	20 (0.60%)	1 (0.69%)	0.590
Postnatal adaptation	101 (2.89%)	98 (2.88%)	3 (2.97%)	0.767	95 (2.83%)	6 (4.14%)	0.311
Birth asphyxia							
Mild	154 (4.40%)	140 (4.12%)	14 (13.86%)	< 0.001	125 (3.73%)	29 (20.0%)	< 0.001
Severe	11 (0.31%)	10 (0.29%)	1 (0.99%)	0.276	10 (0.30%)	1 (0.69%)	0.373
Respiratory distress syndrome	14 (0.40%)	13 (0.38%)	1 (0.99%)	0.337	11 (0.33%)	3 (2.07%)	0.018
Transient tachypnea of the newborn	153 (4.37%)	148 (4.35%)	5 (4.95%)	0.802	142 (4.23%)	11 (7.59%)	0.061
Hypothermia	31 (0.89%)	31 (0.91%)	0 (0.00%)	1.000	29 (0.86%)	2 (1.38%)	0.370
Pneumothorax	8 (0.23%)	8 (0.24%)	0 (0.00%)	1.000	7 (0.21%)	1 (0.69%)	0.287
Desaturation	10 (0.29%)	9 (0.26%)	1 (0.99%)	0.254	10 (0.30%)	0 (0.00%)	1.000
Pneumonia	9 (0.26%)	9 (0.26%)	0 (0.00%)	1.000	9 (0.27%)	0 (0.00%)	1.000
Hypocalcaemia	6 (0.17%)	6 (0.18%)	0 (0.00%)	1.000	5 (0.15%)	1 (0.69%)	0.224
Hypoglycaemia	51 (1.46%)	49 (1.44%)	2 (1.98%)	0.658	48 (1.43%)	3 (2.07%)	0.468
Sepsis	26 (0.74%)	25 (0.74%)	1 (0.99%)	0.534	25 (0.75%)	1 (0.69%)	1.000
Death	5 (0.14%)	5 (0.15%)	0 (0.00%)	1.000	5 (0.15%)	0 (0.00%)	1.000
Other	43 (1.23%)	41 (1.21%)	2 (1.98%)	0.354	40 (1.19%)	3 (2.07%)	0.423
Total	1348 (38.51%)	1267 (37.28%)	81 (80.20%)	< 0.001	1227 (36.57%)	121 (83.45%)	< 0.001

Similarly, neonates with fetal distress (145/3500 cases) exhibited significant increases in neonatal jaundice (30.34%), mild birth asphyxia (20.0%), caput succedaneum (45.52%), cephalohaematoma, (14.48%), subgaleal haematoma (7.59%), with *P* < 0.001 (Table 6).

Neonates born to anaemic mothers (haematocrit < 33%; 622/3500 cases) had a significant rise in neonatal jaundice (15.22%, *P* = 0.003; Table 7). Those with low birth weight (1500–2499 g; 239/3500 cases) presented significant complications such as the need for ventilator support (3.77%), neonatal jaundice (28.45%), hypoglycaemia (7.95%), respiratory distress syndrome (1.67%) and hypocalcaemia (1.26%; Table 7).

**Table 7:** Association of neonatal injuries and morbidities with maternal anaemia and neonatal birth weight (univariate analysis).

	All (N = 3500)	Maternal haematocrit		*P*	Neonatal birthweight (grams)			*P*
		Hct ≤ 33% (n = 622)	Hct > 33% (n = 2831)		Normal (2500–4000) (n = 3233)	Low (> 1500–2499) (n = 239)	High (> 4000) (n = 28)	
Caput succedaneum	291 (8.31%)	46 (7.40%)	244 (8.62%)	0.319	274 (8.48%)	15 (6.28%)	2 (7.14%)	0.481
Petechia	108 (3.09%)	17 (2.73%)	91 (3.21%)	0.532	98 (3.03%)	7 (2.93%)	3 (10.71%)	0.064
Jaundice	507 (14.49%)	66 (10.61%)	431 (15.22%)	0.003	436 (13.49%)	68 (28.45%)	3 (10.71%)	< 0.001
Neonatal injuries								
Bruise	5 (0.14%)	17 (2.73%)	73 (2.53%)	0.827	81 (2.51%)	8 (3.35%)	1 (3.57%)	0.690
Abrasion	90 (2.57%)	9 (1.45%)	55 (1.94%)	0.406	52 (1.61%)	11 (4.60%)	1 (3.57%)	0.003
Cephalohaematoma	79 (2.26%)	8 (1.29%)	70 (2.47%)	0.071	71 (2.20%)	6 (2.51%)	2 (7.14%)	0.207
Scalp laceration	49 (1.40%)	7 (1.13%)	42 (1.48%)	0.494	43 (1.33%)	6 (2.51%)	0 (0.00%)	0.266
Subgaleal haematoma	24 (0.69%)	8 (1.29%)	16 (0.57%)	0.061	23 (0.71%)	0 (0.00%)	1 (3.57%)	0.078
Brachial plexus injury	4 (0.11%)	1 (0.16%)	3 (0.11%)	0.548	4 (0.12%)	0 (0.00%)	0 (0.00%)	0.848
Fractured clavicle	0 (0.0%)	4 (0.64%)	20 (0.71%)	1.000	22 (0.68%)	2 (0.84%)	1 (3.57%)	0.190
Neonatal morbidities								
Ventilatory support	21 (0.60%)	4 (0.64%)	16 (0.57%)	0.772	12 (0.37%)	9 (3.77%)	0 (0.00%)	< 0.001
Postnatal adaptation	101 (2.89%)	20 (3.22%)	79 (2.79%)	0.565	94 (2.91%)	7 (2.93%)	0 (0.00%)	0.657
Birth asphyxia								
Mild	154 (4.40%)	30 (4.82%)	123 (4.34%	0.600	144 (4.45%)	8 (3.35%)	2 (7.14%)	0.562
Severe	11 (0.31%)	2 (0.32%)	8 (0.28%)	0.698	8 (0.25%)	2 (0.84%)	1 (3.57%)	0.002
Transient tachypnea of the newborn	153 (4.37%)	31 (4.98%)	121 (4.27%)	0.435	140 (4.33%)	9 (3.77%)	4 (14.29%)	0.033
Respiratory distress syndrome	26 (0.74%)	3 (0.48%)	8 (0.28%)	0.429	10 (0.31%)	4 (1.67%)	0 (0.00%)	0.005
Pneumothorax	31 (0.89%)	2 (0.32%)	6 (0.21%)	0.641	7 (0.22%)	1 (0.42%)	0 (0.00%)	0.794
Desaturation	8 (0.23%)	1 (0.16%)	9 (0.32%)	1.000	9 (0.28%)	1 (0.42%)	0 (0.00%)	0.890
Pneumonia	10 (0.29%)	2 (0.32%)	7 (0.25%)	0.669	8 (0.25%)	1 (0.42%)	0 (0.00%)	0.849
Hypercalcaemia	9 (0.26%)	3 (0.48%)	3 (0.11%)	0.076	3 (0.09%)	3 (1.26%)	0 (0.00%)	< 0.001
Hypoglycaemia	51 (1.46%)	10 (1.61%)	37 (1.31%)	0.558	31 (0.96%)	19 (7.95%)	1 (3.57%)	< 0.001
Hypothermia	14 (0.40%)	8 (1.29%)	23 (0.81%)	0.257	26 (0.80%)	4 (1.67%)	1 (3.57%)	0.120
Sepsis	25 (0.71%)	7 (1.13%)	19 (0.67%)	0.301	21 (0.65%)	4 (1.67%)	1 (3.57%)	0.044
Death	6 (0.17%)	1 (0.16%)	4 (0.14%)	1.000	3 (0.09%)	2 (0.84%)	0 (0.00%)	0.013
Other	64 (1.83%)	15 (2.41%)	26 (0.92%)	0.002	36 (1.11%)	6 (2.51%)	1 (3.57%)	0.088
Total	1348 (38.51%)	220 (35.37%)	1109 (39.17%)	0.078	1207 (37.33%)	127 (53.14%)	14 (50.00%)	< 0.001

Based on multivariate analysis, factors associated with caput succedaneum were assisted vacuum delivery (AOR 5.18, 95% CI 2.60–10.3, *P* < 0.001) and fetal distress (AOR 1.95, 95% CI 1.02–3.74, *P* < 0.044). Cephalohaematoma was linked to GDMA2 (AOR 11.3, 95% CI 2.96–43.2, *P* < 0.001) and assisted vacuum delivery (AOR 16.5, 95% CI 6.71–40.5, *P* < 0.001). Scalp laceration was correlated with GDMA2 (AOR 10.5, 95% CI 2.28–48.1, *P* = 0.003), assisted vacuum delivery (AOR 6.94, 95% CI 1.85–26.1, *P* = 0.004) and assisted forceps delivery (AOR 10.5, 95% CI 1.08–102.2, *P* = 0.042). Factors associated with abrasion and bruising were assisted forceps delivery (AOR 39.1, 95% CI 11.5–132.5, *P* < 0.001) and preterm delivery (AOR 3.49, 95% CI 1.39–8.72, *P* = 0.008; Table 8).

**Table 8:** Determinants of neonatal cephalic injuries (multivariable analysis).

Factors associated with caput succedaneum (n = 291)	Unadjusted odds ratio (95% CI)	*P*	Adjusted odds ratio (95% CI)	*P*
Parity ≥ 1	0.31 (0.24, 0.40)	< 0.001	0.36 (0.22, 0.57)	< 0.001
Hypertension (HT)				
No	Ref		Ref	
Gestational HT/transient	1.57 (1.01, 2.45)	0.047	1.54 (0.95, 2.49)	0.081
Severe pre-eclampsia	1.63 (0.37, 7.20)	0.521	2.43 (0.47, 12.5)	0.289
Pre-existing HT	–	–	–	–
Surgeon				
Resident and fellow	Ref		Ref	
Staff	0.26 (0.11, 0.64)	0.003	0.42 (0.17, 1.03)	0.058
Medical students and midwives	0.98 (0.59, 1.61)	0.919	0.69 (0.40, 1.20)	0.187
Vaginal delivery				
Spontaneous	Ref		Ref	
Assisted vacuum delivery	10.7 (7.83, 14.5)	< 0.001	5.18 (2.60, 10.3)	< 0.001
Assisted forceps delivery	3.04 (0.66, 14.0)	0.153	1.94 (0.38, 9.97)	0.430
Prolonged second stage of labour	7.86 (5.16, 12.0)	< 0.001	1.17 (0.64, 2.15)	0.603
Fetal distress	11.6 (8.16, 16.6)	< 0.001	1.95 (1.02, 3.74)	0.044*

Factors associated with cephalohaematoma (n = 79)	Unadjusted odds ratio (95% CI)	*P*	Adjusted odds ratio (95% CI)	*P*
Parity ≥ 1	0.58 (0.37, 0.91)	0.017	0.89 (0.35, 2.25)	0.797
Gestational diabetes mellitus (GDM)				
No	Ref		Ref	
GDMA1	1.25 (0.64, 2.44)	0.522	1.18 (0.58, 2.40)	0.649
GDMA2	7.62 (2.19, 26.5)	0.001	11.3 (2.96, 43.2)	< 0.001*
Pre-existing diabetes mellitus	–	–	–	–
Vaginal delivery				
Spontaneous	Ref		Ref	
Assisted vacuum delivery	14.6 (9.12, 23.3)	< 0.001	16.5 (6.71, 40.5)	< 0.001*
Assisted forceps delivery	–	–	–	–
Prolonged second stage of labour	11.9 (6.71, 20.9)	< 0.001	1.12 (0.50, 2.51)	0.776
Fetal distress	9.63 (5.66, 16.4)	< 0.001	0.69 (0.30, 1.58)	0.381
Maternal haematocrit ≤ 33%	1.95 (0.93, 4.07)	0.071	2.17 (0.96, 4.88)	0.061
Underlying disease	2.34 (0.93, 5.92)	0.064	1.47 (0.53, 4.07)	0.458
Maternal weight (kg)	0.99 (0.97, 1.01)	0.185	0.98 (0.96, 1.01)	0.099

Factors associated with scalp laceration (n = 49)	Unadjusted odds ratio (95% CI)	*P*	Adjusted odds ratio (95% CI)	*P*
Gestational diabetes mellitus (GDM)				
No	Ref		Ref	
GDMA1	1.69 (0.79, 3.65)	0.179	1.14 (0.47, 2.75)	0.773
GDMA2	8.22 (1.85, 36.5)	0.006	10.5 (2.28, 48.1)	0.003*
Pre-existing diabetes mellitus	–	–	–	–
Hypertension (HT)				
No	Ref		Ref	
Gestational HT/transient	1.98 (0.78, 5.070	0.152	1.68 (0.61, 4.62)	0.317
Severe pre-eclampsia	–	–	–	–
Pre-existing HT	–	–	–	–
Surgeon				
Residents and fellows	Ref		Ref	
Staff	0.75 (0.18, 3.12)	0.692	1.07 (0.25, 4.50)	0.931
Medical students, midwives	2.60 (1.15, 5.87)	0.022	2.33 (0.98, 5.56)	0.056
Vaginal delivery				
Spontaneous	Ref		Ref	
Assisted vacuum delivery	4.23 (2.08, 8.62)	< 0.001	6.94 (1.85, 26.1)	0.004*
Assisted forceps delivery	7.73 (0.97, 61.4)	0.053	10.5 (1.08, 102.2)	0.042*
Prolonged second stage of labour	4.93 (2.05, 11.9)	< 0.001	1.18 (0.30, 4.65)	0.817
Fetal distress	2.09 (0.74, 5.88)	0.143	0.32 (0.08, 1.25)	0.101

Factors associated with abrasion and bruising (n = 64)	Unadjusted odds ratio (95% CI)	*P*	Adjusted odds ratio (95% CI)	*P*
Vaginal delivery				
Spontaneous	Ref		Ref	
Assisted vacuum delivery	0.54 (0.13, 2.23)	0.393	0.50 (0.12, 2.10)	0.345
Assisted forceps delivery	40.3 (12.4, 130.7)	< 0.001	39.1 (11.5, 132.5)	< 0.001*
Birthweight (grams)				
Normal (2500–4000)	Ref		Ref	
Low (> 1500–2499)	2.95 (1.52, 5.73)	0.001	1.94 (0.88, 4.28)	0.103
High (> 4000)	2.27 (0.30, 17.0)	0.426	2.87 (0.38, 21.8)	0.307
Gestational age at delivery				
< 37 weeks (preterm)	4.97 (2.31, 10.7)	< 0.001	3.49 (1.39, 8.72)	0.008*
≥ 37 weeks (term)	Ref		Ref	
Parity ≥ 1	0.70 (0.43, 1.15)	0.282	0.75 (0.45, 1.26)	0.206

*P < 0.05 means the results are statistically significant.

Factors connected with neonatal jaundice were deliveries after hours (4.00 PM–8.00 AM; AOR 1.32, 95% CI 1.07–1.63, *P* = 0.009), assisted vacuum delivery (AOR 2.22, 95% CI 1.09–4.52, *P* = 0.029), preterm delivery (AOR 3.08, 95% CI 1.92–4.93, *P* < 0.001), maternal anaemia (haematocrit < 33%; AOR 1.36, 95% CI 1.03–1.81, *P* = 0.033) and neonatal low birth weight (AOR 2.11, 95% CI 1.49–2.98, *P* < 0.001; Table 9).

**Table 9:** Determinants of neonatal complications (multivariable analysis).

Factors associated with Jaundice (n = 507)	Unadjusted odds ratio (95% CI)	*P*	Adjusted odds ratio (95% CI)	*P*
Parity ≥ 1	0.69 (0.57, 0.83)	< 0.001	0.81 (0.54, 1.19)	0.282
Hypertension (HT)				
No	Ref		Ref	
Gestational HT/transient	1.49 (1.04, 2.15)	0.031	1.31 (0.89, 1.93)	0.167
Severe pre-eclampsia	1.40 (0.40, 4.95)	0.597	1.23 (0.33, 4.59)	0.760
Pre-existing HT	–	–	–	–
Time of delivery				
Day (8.00 AM–4.00 PM)	Ref		Ref	
Night (4.00 PM–8.00 AM)	1.29 (1.06, 1.58)	0.012	1.32 (1.07, 1.63)	0.009*
Vaginal delivery				
Spontaneous	Ref		Ref	
Assisted vacuum delivery	2.72 (1.99, 3.71)	< 0.001	2.22 (1.09, 4.52)	0.029*
Assisted forceps delivery	–	–	–	–
Gestational age at delivery				
< 37 weeks (preterm)	3.92 (2.61, 5.89)	< 0.001	3.08 (1.92, 4.93)	< 0.001*
≥ 37 weeks (term)	Ref		Ref	
Prolonged second stage of labour	2.22 (0.14, 3.48)	< 0.001	0.89 (0.47, 1.71)	0.726
Fetal distress	2.72 (1.89, 3.93)	< 0.001	1.33 (0.66, 2.67)	0.422
Maternal haematocrit ≤ 33%	1.51 (1.15, 1.99)	0.003	1.36 (1.03, 1.81)	0.033*
Maternal obesity	1.01 (0.99, 1.02)	0.053	1.02 (0.98, 1.03)	0.055
Birthweight (grams)				
Normal (2500–4000)	Ref		Ref	
Low (> 1500–2499)	2.55 (1.89, 3.44)	< 0.001	2.11 (1.49, 2.98)	< 0.001*
High (> 4000)	0.77 (0.23, 2.56)	0.670	0.60 (0.17, 2.05)	0.411

Factors associated with hypoglycaemia (n = 51)	Unadjusted odds ratio (95% CI)	*P*	Adjusted odds ratio (95% CI)	*P*
Hypertension (HT)				
No	Ref		Ref	
Gestational HT/transient	1.10 (0.34, 3.57)	0.875	1.41 (0.41, 4.85)	0.582
Severe pre-eclampsia	10.2 (2.25, 46.2)	0.003	10.1 (1.94, 52.6)	0.006*
Pre-existing HT	–	–	–	–
Birthweight (grams)				
Normal (2500–4000)	Ref		Ref	
Low (> 1500–2499)	8.92 (4.96, 16.0)	< 0.001	7.52 (3.79, 14.9)	< 0.001*
High (> 4000)	3.83 (0.50, 29.0)	0.195	4.92 (0.59, 41.2)	0.142
Surgeon				
Residents and fellows	Ref		Ref	
Staff	2.62 (1.16, 5.92)	0.020	2.18 (0.87, 5.49)	0.097
Medical students, midwives	1.07 (0.33, 3.47)	0.917	1.45 (0.43, 4.83)	0.549
Gestational age at delivery				
< 37 weeks	6.50 (2.97, 14.2)	< 0.001	2.13 (0.83, 5.44)	0.114
≥ 37 weeks	Ref		Ref	

Factors associated with transient tachypnea of the newborn (n = 153)	Unadjusted odds ratio (95% CI)	*P*	Adjusted odds ratio (95% CI)	*P*
Hypertension (HT)				
No	Ref		Ref	
Gestational HT/transient	1.75 (0.99, 3.09)	0.055	1.54 (0.85, 2.78)	0.153
Severe pre-eclampsia	5.30 (1.49, 18.8)	0.010	4.48 (1.23, 16.4)	0.023*
Pre-existing HT	–	–	–	–
Vaginal delivery				
Spontaneous	Ref		Ref	
Assisted vacuum delivery	1.75 (1.01, 3.04)	0.047	1.23 (0.44, 3.47)	0.692
Assisted forceps delivery	–	–	–	–
Gestational age at delivery				
< 37 weeks	1.87 (0.89, 3.92)	0.135	2.28 (1.02, 5.07)	0.044*
≥ 37 weeks	Ref		Ref	
Fetal distress	1.86 (0.98, 3.51)	0.061	1.42 (0.43, 4.77)	0.567
Birthweight (grams)				
Normal (2500–4000)	Ref		Ref	
Low (> 1500–2499)	0.87 (0.44, 1.72)	0.678	0.76 (0.36, 1.60)	0.464
High (> 4000)	3.68 (1.26, 10.8)	0.017	2.73 (0.89, 8.36)	0.078

Factors associated with Asphyxia (n = 165)	Unadjusted odds ratio (95% CI)	*P*	Adjusted odds ratio (95% CI)	*P*
Parity ≥ 1	0.73 (0.53, 0.99)	0.044	0.74 (0.40, 1.39)	0.349
Gestational diabetes mellitus (GDM)				
No	Ref		Ref	
GDMA1	1.46 (0.94, 2.29)	0.096	1.39 (0.87, 2.24)	0.173
GDMA2	3.59 (1.04, 12.3)	0.043	3.64 (1.03, 12.8)	0.044*
Pre-existing diabetes mellitus	–	–	–	–
Prolonged second stage of labour	3.78 (2.13, 6.70)	< 0.001	0.79 (0.37, 1.73)	0.564
Vaginal delivery				
Spontaneous	Ref		Ref	
Assisted vacuum delivery	5.62 (3.79, 8.34)	< 0.001	3.44 (1.36, 8.69)	0.009*
Assisted forceps delivery	–	–	–	–
Fetal distress	1.01 (1.00, 1.03)	0.037	1.02 (1.01, 1.03)	0.013*

Factors associated with ventilatory support (n = 21)	Unadjusted odds ratio (95% CI)	*P*	Adjusted odds ratio (95% CI)	*P*
Gestational age at delivery				
< 37 weeks	13.8 (5.24, 36.3)	< 0.001	5.07 (1.61, 16.0)	0.006*
≥ 37 weeks	Ref		Ref	
Birthweight (grams)				
Normal (2500–4000)	Ref		Ref	
Low (> 1500–2499)	10.5 (4.38, 25.2)	< 0.001	5.79 (2.02, 16.6)	0.001*
High (> 4000)	–	–	–	–
Maternal weight (kg)	0.97 (0.93, 1.01)	0.142	0.99 (0.95, 1.03)	0.636

Factors associated with postnatal adaptation (n = 101)	Unadjusted odds ratio (95% CI)	*P*	Adjusted odds ratio (95% CI)	*P*
Time of delivery				
Day (8.00 AM–4.00 PM)	Ref		Ref	
Night (4.00 PM–8.00 AM)	2.13 (1.32, 3.43)	0.002	2.10 (1.30, 3.39)	0.002*
Gestational diabetes mellitus (GDM)				
No	Ref		Ref	
GDMA1	1.02 (0.11, 1.19)	0.091	1.05 (0.12, 1.24)	0.056
GDMA2	1.56 (0.21, 11.7)	0.668	1.43 (0.19, 10.8)	0.731
Pre-existing DM	–	–	–	–
Vaginal delivery				
Spontaneous	Ref		Ref	
Assisted vacuum delivery	1.78 (0.91, 3.47)	0.092	1.80 (0.92, 3.53)	0.086
Assisted forceps delivery	3.25 (0.42, 25.4)	0.262	3.76 (0.47, 30.0)	0.211

*P < 0.05 means the results are statistically significant.

Neonatal hypoglycaemia was associated with births from mothers with severe pre-eclampsia (AOR 10.1, 95% CI 1.94–52.6, *P* = 0.006) and neonatal low birth weight (AOR 7.52, 95% CI 3.79–14.9, *P* < 0.001; Table 9). Transient tachypnoea of the newborn was linked to births from mothers with severe pre-eclampsia (AOR 4.48, 95% CI 1.23–16.4, *P* = 0.023) and preterm delivery (AOR 2.28, 95% CI 1.02–5.07, *P* = 0.044; Table 9). Neonatal asphyxia was associated with births from mothers with GDMA2 (AOR 3.64, 95% CI 1.03–12.8, *P* = 0.044), assisted vacuum delivery (AOR 3.44, 95% CI 1.36–8.69, *P* = 0.009) and fetal distress (AOR 1.02, 95% CI 1.01–1.03, *P* = 0.013; Table 9). Neonates requiring ventilator support were linked to preterm delivery (AOR 5.07, 95% CI 1.61–16.0, *P* = 0.006) and neonatal low birth weight (AOR 5.79, 95% CI 2.02–16.6, *P* = 0.001; Table 9).

## Discussion

Our study revealed that the overall incidence of neonatal morbidities and complications arising from vaginal delivery was 38.5% (1348/3500 cases). The most frequently observed complications were neonatal jaundice (14.5%, 507/3500 cases) and caput succedaneum (8.3%, 291/3500 cases).

Caput succedaneum refers to scalp oedema that emerges during labour and typically resolves within 48 h post-birth. This condition commonly presents following prolonged engagement of the fetal head in the birth canal or after vacuum extraction. On the other hand, neonatal jaundice or neonatal hyperbilirubinaemia arises from elevated total serum bilirubin levels. It manifests clinically as a yellowish discolouration of the skin, sclera and mucous membranes. Notably, approximately 60% of term and 80% of preterm newborns exhibit clinical jaundice during their first week post-birth^7^. This manifestation is generally a mild, transient and self-limiting condition that resolves without any intervention and is termed ‘physiological jaundice’. Nevertheless, it is crucial to differentiate it from the more severe ‘pathological jaundice’ to ensure early intervention and prevention of significant complications.

Furthermore, caput succedaneum may give rise to compression-related necrotic lesions, potentially leading to long-term scarring and alopecia^8^. The ‘halo scalp ring’ is an annular alopecic circle seen in infants after enduring prolonged or challenging labour. This condition arises due to compression from the bony prominences of the maternal pelvis^9^. Instances of infected caput succedaneum are rare^10^.

Bruises and petechiae were the most frequently observed findings in our study. They are generally self-limiting and commonly appear on the presenting portion of the newborn’s body. A bruise is a risk factor for the development of hyperbilirubinaemia or jaundice. Hence, it is advisable to closely monitor infants with significant bruising to evaluate the potential development of jaundice^11^.

Our research indicated that assisted deliveries using vacuum or forceps were closely linked to the occurrence of scalp lacerations. Additionally, assisted forceps delivery was notably associated with abrasions and bruises. Assisted vaginal deliveries account for 10–15% of births in Canada^12^, Australia^13^ and the United Kingdom^14^. When well-trained physicians conduct such operations, there is a demonstrated association with reduced risks for mothers and newborns^14–16^. However, the shift from assisted vaginal deliveries to caesarean deliveries has reduced opportunities for medical professionals to gain expertise in using vacuum and forceps^12,17,18^. Consequently, it is unsurprising that there have been increasing reports of maternal and neonatal trauma related to assisted vaginal deliveries. This has also intensified concerns regarding the comparative safety of using forceps versus vacuum^19–21^.

In our study, clavicle fractures accounted for 0.7% (25/3500 cases) of the injuries. These fractures were not significantly linked to assisted vaginal deliveries. According to extensive case series, the incidence of clavicle fractures stemming from birth trauma varies between 0.5 and 1.6%^22,23^. Fractured clavicles often correlate with challenging vaginal deliveries due to factors such as assisted delivery, shoulder dystocia, advanced maternal age, and babies large for their gestational age^22,23^. When a clavicle fracture is identified, it is essential to investigate any concurrent brachial plexus injury. In our dataset, the incidence of brachial plexus injury was 0.1% (4/3500 cases), and all instances resulted from spontaneous vaginal delivery. The only acknowledged risk factor for brachial plexus injury is shoulder dystocia, for which no validated predictive or preventative measures exist. Potential mechanisms leading to neonatal brachial plexus palsy encompass stretching/traction, compression, infiltration and oxygen deprivation^24^. Physicians tasked with performing assisted vaginal deliveries must be well acquainted with these mechanisms and be adequately trained to avoid causing injury.

Fetal asphyxia was significantly associated with mothers having GDMA2 in our research, corroborating the findings by Kawakita et al.^25^. This association was more pronounced in pregestational diabetes than in gestational diabetes^25^, although the exact cause remains elusive. The suggested mechanisms for this link are fetal hyperglycaemia and hyperinsulinemia, which might heighten neonatal respiratory morbidity, even in vaginal deliveries.

The predominant neonatal morbidities we observed were hypoglycaemia, hypocalcaemia and hyperthermia, which were significantly related to neonatal low birth weight. These findings are consistent with a study by Chand et al.^26^.

Our research indicated that neonatal birth injuries were significantly correlated with preterm delivery, assisted vaginal deliveries, and babies large for their gestational age. Previous research found that the risk of birth injury was double for infants weighing between 4000 and 4499 g compared to those of average birth weight. The risk was threefold for infants weighing between 4500 and 4999 g and surged to 4.5 times for those weighing more than 5000 g^27^. Another study found a 7.7% incidence of fetal injury in infants weighing over 4500 g^28^. Other studies have emphasised that preterm infants are particularly prone to respiratory complications due to their premature birth, a finding consistent with our observations^29^.

Maternal obesity, defined by a body mass index of > 40 kg/m^2^, increases the risk of birth injuries. This group of parturients often has higher rates of assisted vaginal delivery and an elevated risk of bearing a large-for-gestational-age infant, which can further raise the likelihood of shoulder dystocia^30^. However, our study did not observe a significant incidence of brachial plexus injury.

Interestingly, our findings highlight a significant occurrence of neonatal injuries and morbidities after regular hospital (daytime) hours. US and Austrian national studies indicate that there are heightened risks for adverse maternal and neonatal outcomes during night deliveries^31–33^. However, Yee et al. noted that academic medical centres equipped with round-the-clock specialists produced similar outcomes for pregnant women with postpartum haemorrhage, regardless of the delivery time^34^. Continuous in-house staffing in obstetric units might, therefore, enhance night-time outcomes.

### Implications for clinical practice

In our study, we observed a high incidence of neonatal morbidities and complications, underscoring the importance of healthcare practitioners prioritizing vigilant monitoring of newborns delivered vaginally, particularly those born through assisted methods like vacuum or forceps. Early detection and management of complications such as neonatal jaundice and caput succedaneum are crucial in preventing long-term sequelae.

The study emphasized the significance of ensuring healthcare professionals receive sufficient training and maintain expertise in conducting assisted vaginal deliveries. Continuous education and simulation training are essential for sustaining proficiency in utilizing vacuum and forceps, thus reducing the risks of maternal and neonatal trauma linked with these procedures.

Our research highlights the crucial need for healthcare professionals to receive adequate training and experience in conducting assisted vaginal deliveries. Continuous education and simulation training are vital in maintaining proficiency with vacuum and forceps, thus reducing the risks of maternal and neonatal trauma associated with these procedures.

The notable incidence of neonatal injuries and morbidities following regular hospital hours underscores the necessity of continuous in-house staffing in obstetric units. This staffing approach could enhance outcomes during nighttime deliveries, guaranteeing prompt access to specialized care as necessary.

### Limitations of the study

Our study's reliance on retrospective data from medical records may introduce bias due to incomplete documentation or variations in reporting practices across different healthcare facilities. Additionally, the retrospective nature of the study limits our ability to establish causality between maternal and neonatal factors and the observed complications. The findings of our study may not be generalizable to populations outside of the study setting or to different healthcare systems with varying practices regarding vaginal deliveries and neonatal care.

### Gaps in existing literature and implications for future research

Future research could further explore the comparative safety and efficacy of vacuum versus forceps-assisted vaginal deliveries, specifically focusing on neonatal outcomes and maternal morbidity. Longitudinal studies with larger sample sizes are necessary to offer more robust evidence in this domain.

Further investigation into additional maternal and neonatal factors, such as maternal obesity, maternal age, and birth order, could yield valuable insights into neonatal complications and risk factors. This deeper understanding could aid in risk stratification and the development of targeted interventions to mitigate adverse outcomes.

Additional research is needed to investigate the effects of delivery timing, including daytime versus nighttime deliveries, on neonatal outcomes and maternal health. Prospective studies with standardized data collection protocols and comprehensive risk adjustment are warranted to better comprehend the factors influencing outcomes during various times of the day.

By addressing these limitations and suggesting avenues for future research, the contribution to the ongoing efforts to improve maternal and neonatal care and reduce the incidence of complications associated with vaginal deliveries.

## Conclusions

Neonatal injuries and morbidities were frequently observed in cases involving assisted vacuum delivery, maternal GDMA2, severe-feature pre-eclampsia, maternal anaemia, preterm delivery, and low birth weight. These morbidities were often associated with factors such as a prolonged second stage of labour, fetal distress, maternal obesity, and infants being large for their gestational age. Notably, most of these morbidities manifested during night-time.

## Data Availability

The datasets used or analysed during the current study available from the corresponding author on reasonable request.
